# The use of Transcollation Technology for Video-Assisted Thoracic Surgery lobectomy

**DOI:** 10.1186/s13019-020-01230-y

**Published:** 2020-07-28

**Authors:** Cecilia Menna, Camilla Poggi, Claudio Andreetti, Anna Maria Ciccone, Alberto Emiliano Baccarini, Giulio Maurizi, Antonio D’Andrilli, Camilla Vanni, Roberto Cascone, Alfonso Fiorelli, Mario Santini, Federico Venuta, Erino Angelo Rendina, Mohsen Ibrahim

**Affiliations:** 1 Division of Thoracic Surgery, Sapienza University of Rome, AOU Sant’Andrea, Rome, Italy; 2 Division of Thoracic Surgery, University of Campania Luigi Vanvitelli, Piazza Miraglia, 2, I-80138 Naples, Italy; 3 Division of Thoracic Surgery, Sapienza University of Rome, AOU Policlinico Umberto I, Rome, Italy

**Keywords:** Transcollation technology, Bipolar sealer, VATS lobectomy, Hilar dissection, Blood loss reduction

## Abstract

**Background:**

Video-Assisted Thoracic Surgery (VATS) lobectomy is now considered the preferred approach at many centers for early stage lung cancer. However, it needs an adequate learning curve, and it may be challenging in non-expert hands. The aim of this study was to evaluate the effectiveness of Transcollation Technology over Traditional Electrocautery to perform hilar and mediastinal dissection during VATS lobectomy.

**Methods:**

This is a single-center retrospective study including consecutive patients undergoing VATS lobectomy for lung cancer. Patients were divided in two groups based on whether Transcollation Technology (TT Group) or Traditional Electrocautery (TE Group) was used for hilar and mediastinal lymphadenectomy. Operative time and surgical outcome, including number of transfusions, length of chest drainage, length of hospital stay, morbidity and mortality were registered, and the inter-group differences were statistically analyzed.

**Results:**

53 patients were included in the final analysis. The TT Group (*n* = 24) compared to the TE Group (*n* = 29) showed significant shorter operative time (75.2 ± 25.8 min versus 98.1 ± 33.3 min; *p* = 0.023), and reduction of length of chest tube stay (4.7 ± 0.8 days vs. 6.8 ± 1.1 days, *p* = 0.013) and length of hospital stay (5.3 ± 1.9 days vs. 6.8 ± 1.1 days, *p* = 0.007). No intraoperative or major postoperative complications were observed in either groups.

**Conclusions:**

Transcollation Technology represents a valid alternative to standard electrocautery instruments during VATS lobectomy. It contributes to reduce the operative time and length of hospital stay. Further larger prospective studies are required to confirm our data.

## Introduction

Surgery is the treatment of choice for early stage Non-Small Cell Lung Cancer (NSCLC), and lobectomy still remains the preferred approach with 5-year survival rate higher than other surgical procedures [1]. Over the years, an increasing number of lobectomies have been performed by Video-Assisted Thoracic Surgery (VATS) due to a reduced morbidity and mortality, better cosmetic results and similar oncological outcome [2, 3]. During the last 5 years, at many centers uniportal VATS has replaced the triportal approach; it has been used both for minor and major procedures [4–7]. However, VATS lobectomy needs an adequate learning curve; not only the technical details of the resection, but also hilar and mediastinal dissection may be challenging in not expert hands [8]. Standard electrocautery is usually used in open procedures; however their use may be unfeasible during VATS due to the size mismatch between the surgical incisions and the caliber of the instruments. Furthermore, conventional electro-coagulation systems may damage the surrounding structures due to lateral heat dispersion with immediate or delayed complications as esophageal injury, phrenic or recurrent laryngeal injury and other potentially life-threatening complications; this might happen both with the open and the VATS approach. Alternative energy sources, as ultrasonic devices or electrothermal bipolar sealing devices have been used in VATS lobectomy, but their use has been limited to the division of minor pulmonary artery vessels, and lymphadenectomy [9, 10]. Transcollation technology (TT) is a disposable bipolar sealing device that uses a radiofrequency coagulation system providing hemostatic soft tissue and bone sealing and delivering of saline solution that produces a localized cooling without scarring. In orthopedic surgery, its use significantly reduced intraoperative blood loss, but no data are reported for VATS procedures [11–13].

The aim of this study was to compare TT over traditional electrocautery to perform hilar and mediastinal dissection during VATS lobectomy.

## Materials and methods

### Study design

All patients undergoing VATS lobectomy for lung cancer (November 2016–October 2018) were retrospectively included in this study. Exclusion criteria were: coagulative disorders, conversion to thoracotomy, previous thoracic or mediastinal surgery, intraoperative use of other hemostatic devices. Patients were divided in two groups; the TT Group with hilar and mediastinal lymphadenectomy performed using TT technology, and the TE Group using Traditional Electrocautery. The choice of using TT or TE was not randomized, however TT was routinely used from January 2018. Assessment of the operative time and surgical outcomes were the primary and secondary end-points of the study respectively. The study was approved by the Local Ethics Committee (Sapienza University of Rome, Italy. Code number: 105 SA_2016). All patients signed a written informed consent informing also that data could be anonymously used for scientific purposes. All surgical procedures were performed by surgeons with a wide expertise in VATS procedures.

### Study population

All patients with NSCLC were considered as candidate for lobectomy based on standard oncological and functional criteria. Demographic data, preoperative comorbidities stratified according to the Charlson index, lung function test, histology, pathological stage, surgical approach (uniportal VATS versus tri-port VATS), procedure time (minutes), daily chest drainage amount (mL), length of chest drainage, length of hospital stay (LOHS) (days), postoperative morbidity and mortality were recorded for each patient.

### TT device

The Aquamantys® Endo VS 8.7R (Medtronic, Minneapolis, MN, USA) is an instrument with double electrodes at the tip. The length of the disposable sealer provides the ability to perform thoracoscopic procedures. The hand piece is equipped with an on/off switch that simultaneously activates both RF energy and saline flow. A saline bag is connected to a pump on the device that pushes the saline through a tube to the hand piece.

### Operative technique

**Fig. 1:**
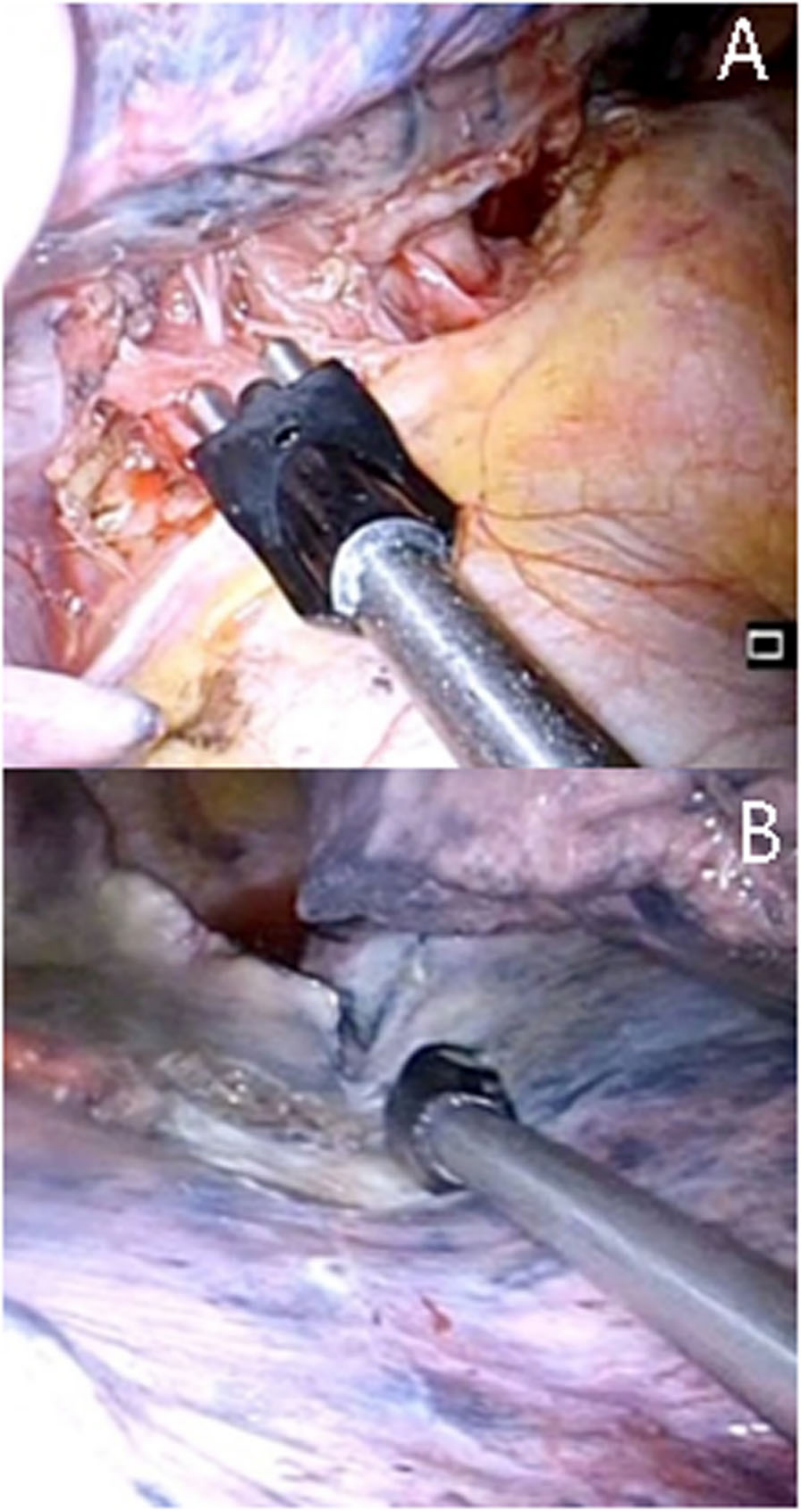
Part **a** showed the dissection of mediastinal pluera and the isolation of hilar structures with Transcollation Technology hilar dissection. Part **b** showed the dissection of thin fissure with Transcollation Technology

Surgery was performed under general anesthesia and single-lung ventilation with the patient in the lateral decubitus position. Both the surgeon and assistant stood anteriorly facing the patient and a second assistant stood posteriorly. For uniportal VATS a single incision of approximately 4–5 cm was performed at the 5th intercostal space along the anterior axillary line, anterior to the latissimus dorsi muscle (muscle-sparing technique). In cases of triportal VATS, the procedure was performed through an anterior approach and two surgical incisions. The first surgical incision (1 cm) for the thoracoscope was performed at the 7th or 8th intercostal space along the anterior axillary line. The second incision was performed anteriorly, at the 5th intercostal space (4 cm), and was used for hilar dissection and removal of the lobe at the end of the operation. The third incision (1 cm) was performed at the 6th intercostal space along the posterior axillary line. Trocars or rib retractors were not used. A soft tissue separator was used. When uniportal VATS was performed, the video-thoracoscope (5 mm 30° optic) and multiple VATS instruments were inserted simultaneously. In order to avoid the overlap of multiple instruments that may strike the tip of the thoracoscope, and result in shaky movements on the video monitor, the scope was placed at the posterior angle of the incision. Long curved instruments were used to allow easy simultaneous insertion of two or three devices. No additional skin incisions were performed. Hilar structures were approached anteriorly following the sequence for VATS lobectomy. Mobilization of pulmonary vessels was performed using the TT device to isolate and prepare the pulmonary vein and the pulmonary artery with their branches (Fig. 1/A). Vessels were mechanically stapled individually, along with the lobar bronchus and the pulmonary fissures (ECHELON ENDOPATH, Ethicon Endo-Surgery, Norderstedt, Germany). The pulmonary artery was dissected at the hilum and within the fissure (Fig. 1/B). The fissure was stapled as a final step. Simultaneously, hilar lymph nodes (11R station) were divided and dissected using the TT device. Dissection of mediastinal lymph nodes was performed. No special caution to preserve the recurrent laryngeal nerve was needed in the dissection of the stations 4 and 3 nodes, since the TT device does not damage or char nerves. Pulmonary ligament dissection was performed with the TT device. The resected lobe was retrieved using a specimen bag (ENDOCATCH, Covidien, La Ciotat, France) through the same incision. Systematic mediastinal lymph node dissection was routinely performed. At the end of the operation, a 24 Fr chest tube was inserted at the posterior extremity of the thoracotomy. Pain management was ensured with a multilevel intercostal nerve block using ropivacaine (7.5 mg diluted in 20 ml of saline), 4 ml for each intercostal space, including the intercostal level of the incision and one level above and below. Patients received oral paracetamol (1 g 4 times per day) and oral Ketorolac (20 mg) depending on the clinical need.

### Statistical analysis

Data are expressed as mean and standard deviation for quantitative variables and number and percentage for qualitative variables. Using published data [14], the mean operative time for patients undergoing VATS lobectomy was calculated to be 180 min (standard deviation: 23 min). We felt that a 20% reduction in operative time would be a clinically significant difference that might change operative management of future patients. With a power of 0.80 and alpha of 0.05, this required 21 patients in each group. Chi-square test was used to assess difference between qualitative variables while t-test calculated difference between quantitative data. A value of *p* < 0.05 was considered statistically significant. MedCalc® statistical software Version 12.4.0 was used for analysis.

## Results

During the study period 60 patients underwent VATS lobectomy. Of these, 7 patients were excluded due to coagulative disorders (*n* = 2); intraoperative use of other hemostatic agents (*n* = 5), thus 53 patients were included in the final analysis. The mean age of study population was 63.7 ± 4.9 years old. Most of the patients were male (66%), with stage I tumors (62%), and adenocarcinoma (75%) as histological type. Lobectomy was performed using triportal or uniportal VATS in 28 (53%), and 25 (47%) cases respectively. The TT and TE group included 24 and 29 patients respectively. As listed in Table 1, the two groups were well matched, and no significant differences were found in demographic data, pre-operative comorbidities, lung function, stage, histology and VATS strategy.

**Table 1:** Clinical characteristics of study population

Variables	All patients	TT Group	TE Group	p
Number	53	24	29	–
Age	63.7 ± 4.9	64.2 ± 3.9	62.1 ± 4.5	0.74
Gender (male)	35 (66%)	16 (67%)	19 (65%)	0.85
BMI	25 ± 8.1	24.9 ± 2.8	25.1 ± 3.1	0.65
Charlson index	2.89 ± 0.7	2.8 ± 0.3	2.77 ± 0.3	0.69
Lung Function				
• ppoFEV1%	85.9 ± 3.9	85 ± 4.9	86 ± 3.5	0.87
• ppoDLCO%	82.7 ± 6.9	81.7 ± 8.3	82 ± 6.5	0.75
Pathologic Stage				
• I	33 (62%)	15 (61%)	18 (69%)	0.82
• II	11 (21%)	5 (31%)	6 (22%)	0.98
• III	9 (17%)	4 (8%)	5 (9%)	0.95
Histology				
• Adenocarcinoma	40 (75%)	19 (79%)	21 (72%)	0.57
• Squamous cell carcinoma	11 (21%) 2 (4%)	5 (21%) 0	6 (21%) 2 (7%)	0.98 0.19
• Others				
Surgery				
• Uniportal- VATS	25 (47%)	11 (46%)	14 (48%)	0.86
• Triportal VATS	28 (53%)	13 (54%)	15 (52%)	0.86

*Abbreviations*: *TT Group* transcollation technology group, *TE Group* traditional electrocautery group, *BMI* body mass index*, FEV1* forced expiratory volume in 1 s, *DLCO* carbon monoxide *diffusing* capacity, *VATS* video-assisted thoracic surgery

The results were summarized in Table 2. The mean operative time was 75.2 ± 25.8 min in the TT Group versus 98.1 ± 33.3 min in the TE Group (*p* = 0.023). Only 1 patient (4.2%) received postoperative blood transfusion in TT Group compared to 3 patients (10.3%) in the TE Group (*p* = 0.40). The daily chest drainage output was significantly lower in TT than in TE group during the entire post-operative course. The mean duration of chest tube placement was shorter in the TT Group compared to TE Group (4.7 ± 0.8 vs. 6.8 ± 1.1 days, *p* = 0.013), as well as the LOHS (5.3 ± 1.9 days vs. 7.8 ± 1.1 days, *p* = 0.007). No intraoperative complications were observed in both groups. One patient in TT Group, and 3 patients in TE group presented air leaks more than 5 days (4.2% versus 10.3%; *p* = 0.40) that spontaneously resolved.

**Table 2:** Peri-operative surgical outcome between two study groups

Variables	TT Group (n = 24)	TE Group (n = 29)	p
Operative time (minutes)	75.2 ± 25.8	98.1 ± 33.3	0.023
Blood transfusion	1 (4.2%)	3 (10.3%)	0.40
Daily Chest drainage output (mL)			
• POD 1	200 ± 38	300 ± 55	0.02
• POD 2	250 ± 43	350 ± 69	0.01
• POD 3	200 ± 27	380 ± 71	0.008
Length of Chest drainage stay (days)	4.7 ± 0.8	6.8 ± 1.1	0.013
Length of hospital stay (days)	5.3 ± 1.9	7.8 ± 1.1	0.007
Air leaks ≥5 days	1 (4.2%)	3 (10.3%)	0.4

## Discussion

VATS lobectomy is becoming the approach of choice in early stage NSCLC, this is due to the clear advantages in controlling postoperative pain, reducing LOHS and postoperative morbidities and mortality [2–7]. However, VATS lobectomy requires specific skills. The isolation of hilar structures and lymph node dissection remain demanding procedures for surgeons at the beginning of their learning curve; they may be associated with an increased risk of bleeding and lymphatic leakage, particularly after induction therapy or dissection of pleural adhesions. The Transcollation Technology is an innovative system that combines RF energy and saline instillation to seal soft tissues and bone. It has been successfully used for the management of pneumothorax [15], but its role in VATS lobectomy has never been assessed.

Our study showed that TT revealed several advantages compared to TE with improved clinical outcomes. The length of the device, and its small diameter allowed to perform easily all surgical manoveures through the ports, with a significant reduction of the operative time. The consequent reduction of time of anesthesia allowed immediate extubation, and early recovery of respiratory function; this may be crucial in COPD patients. The bipolar design of TT eliminated the need for a grounding pad, provided hemostatic sealing while reducing char, controlled depth of penetration, and limited the thermal spread. These proprieties resulted in a safe dissection, and a reduction of injury of adjacent structures as (i) esophagus or vagus nerve during lymph node dissection at stations 7, 8 or 9; (ii) laryngeal nerve during lymph nodes dissection at stations 3 and 4; (iii) phrenic nerve, left main pulmonary artery or aorta during lymph nodes during lymph nodes dissection at stations 5 and 6; (iv) stellate ganglion and/or brachial plexus during dissection of apical tumor [16]. Furthermore, the minimal surgical trauma reduced the lymphatic leakage and postoperative tissue reaction. Hence, the significant reduction in daily chest drain output allowed an early removal of chest drainage, resulting in a lower length of hospital stay. By contrast, airleaks did not have a significant impact of length of chest drainage. In fact, only 4 patients (one in the TE Group and 3 in the TT group; *p* = 0.4) presented air leaks for more than 5 days, that spontaneously resolved within the 7th (three cases) or 8th (one case) post-operative day.

The dissection and vessel divisions were carried out with a single instrument without the need to use multiple instruments with significant advantages especially during uniportal VATS, where the introduction of multiple instruments through the single incision increases the trauma to the intercostal nerve.

From an economical point of view, TT (average € 300,00/hand piece) is more expensive than TE (average € 85,00). The mean required reduction in operating room time to overcome the increased costs of approximately € 100 varies between 20 and 30 min [17]. As the use of TT was associated with a mean reduction in operating room time of 23 min, this data alone cannot offset the higher cost of TT. However, in Italy the mean daily hospital cost per patient is € 650,00. Since TT presented a significant reduction in length of hospital stay (mean reduction 2.5 days), it resulted in a reduction of hospital cost of € 1.625. Thus, the saving cost of operating room time in addition to that of hospital stay might well justify the additional costs of TT over standard electro-coagulation device. In addition, the 24 min reduction in mean operative time per operation may allow to attend more operations per month, especially in high volume center. It will result in further economic advantages especially in this current COVID-19 pandemic where institutions have lost a lot of income.

The retrospective nature, the lack of standardization, and the small simple size of the study do not allow to draw definitive conclusions. The procedures were performed by different surgeons and thus the different experience could play a role in operative time results. Yet, from a technical point of view we compared two different electrosurgical instruments as standard electrocautery (a monopolar cautery using current) versus TT (a bipolar sealing device using Radio Frequency). Since different advanced energy devices (i.e. LigaSure® vessel sealing system, Harmonic® ultrasonic energy etc.…) are available for thoracic surgery procedure also in the same hospital, in the future it may be useful to compare the propriety of these instruments to define whether one instrument is better than another to attend VATS lobectomy.

## Conclusions

TT represents a valid alternative to standard electrocautery instruments during VATS lobectomy. The reduction of operative time and LOHS translated in a reduction of health care costs that overcome the initial cost of the instrument. In addition, the reduction of patient exposure to general anesthesia, and its potential harmful effects may be particularly important for COPD patients. Our preliminary results should be corroborated by future, prospective, randomized studied before recommending the widely use of TT for VATS lobectomy.

## Data Availability

Not applicable.

## Abbreviations

VATS: Video-Assisted Thoracic Surgery
NSCLC: Non-Small Cell Lung Cancer
TT: Transcollation technology
RF: Radiofrequency
TE: Tradictional Electrocautery
LOHS: Length of hospital stay
